# Vascular Kv7 channels control intracellular Ca^2+^ dynamics in smooth muscle

**DOI:** 10.1016/j.ceca.2020.102283

**Published:** 2020-12

**Authors:** Yuan-Ming Tsai, Frederick Jones, Pierce Mullen, Karen E. Porter, Derek Steele, Chris Peers, Nikita Gamper

**Affiliations:** a School of Biomedical Sciences, Faculty of Biological Sciences, University of Leeds, Leeds, LS2 9JT, United Kingdom; b Division of Thoracic Surgery, Department of Surgery, Tri-Service General Hospital, National Defence Medical Centre, Taipei 11490, Taiwan; c Leeds Institute of Cardiovascular and Metabolic Medicine, Faculty of Medicine and Health, University of Leeds, Leeds, LS2 9JT, United Kingdom

**Keywords:** AVP, Arginine vasopressin, Ca^2+^, Calcium, ER, Endoplasmic reticulum, GPCRs, G protein coupled receptors, IP_3_, Inositol trisphosphate, IMA, Internal mammary artery, [Ca^2+^]_i_, Intracellular calcium concentration, *E*_m_, Membrane potential, PIP_2_, Phosphatidylinositol 4,5-bisphosphate, PLC, Phospholipase C, RT-PCR, Real-Time Polymerase Chain Reaction, RyR, Ryanodine receptor, VSMCs, Vascular smooth muscle cells, VGCCs, Voltage-gated Ca^2+^ channels, Kv7, Retigabine, Vasopressin, Calcium, Vascular smooth muscle cell, T-type Ca^2+^channels, Phospholipase C

## Abstract

Voltage-gated Kv7 (or KCNQ) channels control activity of excitable cells, including vascular smooth muscle cells (VSMCs), by setting their resting membrane potential and controlling other excitability parameters. Excitation-contraction coupling in muscle cells is mediated by Ca^2+^ but until now, the exact role of Kv7 channels in cytosolic Ca^2+^ dynamics in VSMCs has not been fully elucidated. We utilised microfluorimetry to investigate the impact of Kv7 channel activity on intracellular Ca^2+^ levels and electrical activity of rat A7r5 VSMCs and primary human internal mammary artery (IMA) SMCs. Both, direct (XE991) and G protein coupled receptor mediated (vasopressin, AVP) Kv7 channel inhibition induced robust Ca^2+^ oscillations, which were significantly reduced in the presence of Kv7 channel activator, retigabine, L-type Ca^2+^ channel inhibitor, nifedipine, or T-type Ca^2+^ channel inhibitor, NNC 55-0396, in A7r5 cells. Membrane potential measured using FluoVolt exhibited a slow depolarisation followed by a burst of sharp spikes in response to XE991; spikes were temporally correlated with Ca^2+^ oscillations. Phospholipase C inhibitor (edelfosine) reduced AVP-induced, but not XE991-induced Ca^2+^ oscillations. AVP and XE991 induced a large increase of [Ca^2+^]_i_ in human IMA, which was also attenuated with retigabine, nifedipine and NNC 55-0396. RT-PCR, immunohistochemistry and electrophysiology suggested that Kv7.5 was the predominant Kv7 subunit in both rat and human arterial SMCs; *CACNA1C* (Cav1.2; L-type) and *CACNA1 G* (Cav3.1; T-type) were the most abundant voltage-gated Ca^2+^ channel gene transcripts in both types of VSMCs. This study establishes Kv7 channels as key regulators of Ca^2+^ signalling in VSMCs with Kv7.5 playing a dominant role.

## Highlights

• Inhibition of Kv7 channels induce Ca^2+^ signals through L- and T-type VGCCs in rat and human vascular smooth muscle cells.
• Kv7.5 is functionally the most important subunit to mediate these effects.
• Phospholipase C mediated Kv7 inhibition is likely to contribute to Ca^2+^ oscillations induced in VSMCs by vasopressin.
• Kv7 activator, retigabine, strongly suppresses [Ca^2+^]_i_ oscillations induced by vasopressin or direct Kv7 channel inhibition.

## Graphical abstract

**Figure:**
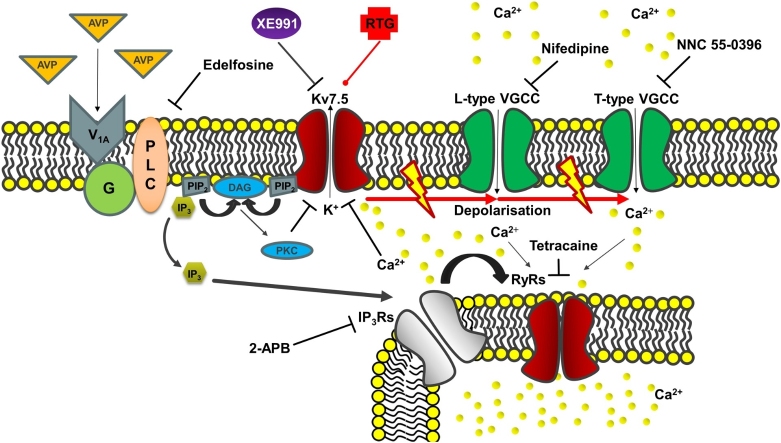

## 1 Introduction

Vascular tone is actively regulated by vasoactive stimuli which control the contractility of vascular smooth muscle cells (VSMCs). The main mechanisms of such control operate via the intracellular calcium (Ca^2+^) signals orchestrating the contraction. Thus, depolarisation of VSMCs membrane potential (*E*_m_*)* results in the opening of voltage-gated Ca^2+^ channels (VGCCs), predominantly L-type [1], while activation of G protein coupled receptors (GPCRs) can induce release of Ca^2+^ from the sarco/endoplasmic reticulum (SR/ER) [2]. Voltage-gated potassium channels (Kv) represent a primary effector system for adjusting the resting *E*_m_ in VSMCs and other cell types [3,4]. Some of these Kv channels are partially open in resting VSMCs and stabilise the resting *E*_m_ at negative voltages to prevent the opening of VGCCs. Inhibition of these Kv channels in VSMCs results in depolarisation which, in turn, may lead to VGCCs activation and, hence, vasoconstriction [5]. To date, the functional roles of some Kv channels in smooth muscle have been examined, especially the Kv1, Kv2 and Kv7 families [[6], [7], [8], [9]]. Furthermore, several vascular diseases such as hypertension, diabetes and atherosclerosis were shown to be associated with the abnormal function or expression of Kv channels [10]. Yet, the exact role of specific Kv channels in the intracellular Ca^2+^ dynamics and in the hormonal control of such dynamics has not been fully elucidated.

One Kv channel subfamily, the Kv7/KCNQ channels, has attracted considerable attention for their role in the control of vascular tone (reviewed in [8,11]). Five members of the family (Kv7.1 to Kv7.5) are widely expressed in excitable and some non-excitable (e.g. epithelial) cells [12]. These channels have a very negative activation threshold (negative to −60 mV), slow kinetics and no inactivation, which allows them to exert a strong ‘clamp’ over the *E*_m_ of cells [6,13]. Kv7.1 subunit is mostly expressed in cardiac and epithelial tissues while Kv7.2-Kv7.5 subunits were long considered to be mostly neuronal [14], yet, recent evidence suggest that several Kv7 subunits are expressed in VSMCs with Kv7.5 considered to be the major subunit [15,16]. The activity of all Kv7 channels depends on the presence of phosphatidylinositol 4,5-bisphosphate (PIP_2_) in the plasma membrane, which is thought to stabilise the channel in the open state [[17], [18], [19]]. Many GPCRs, including the receptors for vasoactive peptides vasopressin, angiotensin II and bradykinin can inhibit Kv7 channels in a well-established signalling cascade which includes activation of phospholipase C (PLC) by Gα_q/11_ G protein. PLC then hydrolyses PIP_2_ to inositol trisphosphate (IP_3_) and diacylglycerol (DAG), with the depletion of membrane PIP_2_ being a major factor mediating the suppression of channel activity with other contributors being intracellular Ca^2+^ and PKC, reviewed in [12,20]. As a general rule, such receptor-mediated Kv7 channel inhibition depolarises the cell and can trigger action potential firing and contraction [6,15,21].

Arginine vasopressin (AVP) is a nonapeptide hormone synthesised in the hypothalamus, which is an essential regulator of the body’s osmotic balance, blood pressure, sodium homeostasis, and kidney functioning [22]. In the vasculature AVP acts via the V1AR receptor and induces vasoconstriction in VSMCs by increasing intracellular Ca^2+^ concentration ([Ca^2+^]_i_) via the influx of Ca^2+^ from L-type Ca^2+^ channels [23]. AVP is an effective therapy to treat patients with vasodilatory shock or intraoperative hypotension [22] and elevated local AVP concentrations were shown to be involved with the maintenance of vasospasm [9]. Vasoactive hormones, including AVP, have been shown to target the activity of vascular Kv7 channels to leverage vascular tone and produce vasoconstriction [8,11,24]. Thus, a previous study demonstrated that at a physiological concentration (100pM) AVP could suppress the Kv7.5 channel currents via PKC activation to depolarise *E*_m_ in A7r5 rat aortic smooth muscle cells [15]. However, the mechanisms of action and relationship between the physiological concentrations of AVP, Kv7 channel activity and [Ca^2+^]_i_ in VSMCs still remain to be clarified [25].

The clinical use of AVP has increased significantly in recent years [26], but its administration could cause vasospasm, which becomes a danger, for instance, when treating refractory vasodilatory shock during bypass surgery [27]. On the other hand, retigabine, a Kv7 activator with anti-epileptic and analgesic properties [6,28], attenuated the basilar artery vasospasm in rats with subarachnoid haemorrhage [9]. Understanding the role of Kv7 channels in controlling [Ca^2+^]_i_ in VSMCs could identify novel approaches for the treatment of cardiovascular disease, including pulmonary hypertension. In the present study, we examined the influence of Kv7 channel activity on [Ca^2+^]_i_ in rat and primary human arterial SMCs.

## 2 Materials and methods

### 2.1 Culture of rat A7r5 cells and human internal mammary arterial VSMCs

Rat A7r5 VSMCs derived from rat thoracic aorta were obtained from the European Collection of Cell Cultures. The A7r5 cell line retains many characteristics of VSMCs, which provides a convenient model system to study mechanisms of Ca^2+^ regulation [29]. Human VSMCs were isolated from the internal mammary artery (IMA) of patients undergoing coronary artery bypass graft (CABG) surgery following ethical permission and informed patient consent. All patients had coronary artery disease without other comorbidities. Specimens were transported to the laboratory under sterile conditions and immediately prepared for culture with a method described previously [30,31]. Vessels were opened longitudinally after removal of adventitia and endothelium. Segments of artery were immersed in 2 mL of Dulbecco’s Modified Eagle Medium (DMEM; Gibco Life Sciences, UK) containing 10 % Fetal Bovine Serum (FBS; Biosera, UK). This was then chopped into fragments around ∼ 1 mm^2^ in size. The artery fragments along with media were then transferred to a culture flask at 37 °C in a humidified incubator with 95 % air and 5 % CO_2_. Rat A7r5 VSMCs were cultured in 75 cm^2^ culture flasks in DMEM containing 10 % FCS (Gibco, UK) at 37 °C (95 % air, 5 % CO_2_). Upon reaching approximately 80 % confluence, the monolayers were subcultured using trypsin-EDTA into 25 cm^2^ culture flasks for Real-Time Polymerase Chain Reaction (RT-PCR) or onto glass coverslips in 24-well tissue culture plates for measurement of [Ca^2+^]_i_ and *E*_m_ by microfluorimetry. For fluorescence measurements, confluent cell monolayers grown on glass coverslips were used 2–5 days after plating.

### 2.2 Fluorescence Ca^2+^ and *E*_m_ imaging

Cells were plated on circular glass coverslips (10 mm, thickness 0) in 24-well plates and kept in a humidified incubator (37 °C; 95 % air; 5 % CO_2_). Once the cell monolayer was confluent, the coverslips were transferred to a 35 mm petri dish and washed with the Ca^2+^ containing buffer twice. The Ca^2+^ containing buffer was composed of (in mM) 135 NaCl, 5 KCl, 1.2 MgSO_4_, 2.5 CaCl_2_, 5 HEPES, and 10 glucose (pH7.4 with NaOH). The osmolality was adjusted to 300 mOsm with sucrose. The cells were loaded with 4 μM Fura 2-AM (Sigma-Aldrich, UK) dissolved in Ca^2+^ containing buffer without pluronic acid for 40 min in the dark at 37 °C. Loading solution was pipetted out and the cells were washed with Ca^2+^ containing buffer. Coverslips were then incubated in Ca^2+^ containing buffer to de-esterify for 10 min. For simultaneous recordings of Ca^2+^ transients and action potentials, rat A7r5 cells were also loaded with the voltage-sensitive dye FluoVolt (Thermo Fisher Scientific, UK) in Ca^2+^ containing buffer supplemented with the dye (1:1000), PowerLoad Concentrate (1:100) and Background Suppressor (1:10) in the dark at 37 °C. Before recording, each coverslip was broken into fragments with a diamond pencil, and fragments were then transferred to a perfusion chamber. Cells were constantly perfused with standard bath solution at 2−3 ml/min using gravity perfusion system via lengths of Tygon tubing (2.5 mm outside diameter, 0.83 mm inside diameter; Merck, UK). The composition of standard bath solution was (in mM): 135 NaCl, 5 KCl, 1.2 MgSO_4_, 2.5 CaCl_2_, 5 HEPES, and 10 glucose (pH7.4 with NaOH); the osmolality adjusted to 300 mOsm with sucrose. The 50 mM K^+^ bath solution was made with isotonic Na^+^ substitution; all drugs were applied in standard bath solution via the perfusion system. Intracellular Ca^2+^ levels were measured ratiometrically using a Cairn Research ME-SE Photometry system (Cairn Research, Kent, UK). Changes in [Ca^2+^]_i_ were evaluated as the ratio of intensities of fluorescence emitted at 510 nm following alternating excitation at 340 and 380 nm (F340, F380) using a monochromator. For measurement of *E*_m_, cells were sequentially excited at 340, 380 and 488 nm (Fura 2-AM and FluoVolt loaded samples). Acquisition Engine 1.6.1 software was used to record and analyse the traces. Magnitude of changes in [Ca^2+^]_i_ was calculated either as peak F340/F380 ratio or as the area under the curve (relative to the basal level or fist spike). Change of *E*_m_ was presented as ΔF/F_0_ in cells.

### 2.3 Endpoint and RT-PCR

To assess the expression of genes coding for Kv7 and VGCC channel subunits, Real-Time Polymerase Chain Reaction (RT-PCR) was used. Cells were cultured in 25 cm^2^ culture flasks and harvested using 1 mL trypsin, centrifuged with 600 *g* for 6 min. Total RNA was extracted from the pelleted cells using the Aurum Total RNA Isolation Protocol (Bio-Rad, Hemel Hempstead, UK). A cDNA template was generated using the iScript cDNA synthesis instructions (Bio-Rad, Hemel Hempstead, UK) with 1 μg RNA. End-point PCR and RT-PCR were performed using TaqMan gene expression assays (Thermo Fisher Scientific, UK). The probes used in this study are listed in the Supplemental Table I. All samples consisted of 1 μl of cDNA and 19 μl of RT-PCR reaction mix (as per manufacturer’s instructions). Quantitative analysis of mRNA expression was determined by using a CFX Connect System (Bio-Rad, UK). The reaction profile was 2 min at 50 °C, 10 min at 95 °C, 15 s at 95 °C for 50–60 cycles, then 1 min at 60 °C. PCR amplification products were separated by the 2.0 % agarose gel electrophoresis with gels containing SYBR safe DNA stain (Invitrogen, UK). Quantitative analysis of mRNA expression was carried out using a CFX Connect System (Bio-Rad, UK), and relative gene expression calculated using the 2^−ΔΔCt^ method with hypoxanthine phosphoribosyltransferase 1 (*Hprt1*) as the housekeeper gene. Statistical analysis was performed on the ΔCt values [32].

### 2.4 Immunofluorescence

Cells were plated onto 24-well culture plates containing circular glass coverslips (10 mm, thickness 0) and kept in a humidified incubator at 37 °C in 95 % air and 5 % CO_2_. The cell layers were washed twice with phosphate buffer saline (PBS) for 5 min at room temperature and then fixed by immersing in 4 % paraformaldehyde (PFA; Sigma, UK) for 10 min. The fixed cells were washed with PBS for 3 times each and then blocked by immersing in 5 % normal donkey serum in PBS containing 0.05 % Tween 20 and 0.25 % Triton X-100 for 30 min to permeabilise the cells. Blocking buffer was removed prior to addition of primary antibody and coverslips were then treated with the primary antibodies against Kv7.1 (anti-Mouse 1:500; Santa Cruz), Kv7.2 (anti-Mouse 1:500; Santa Cruz), Kv7.3 (anti-Rabbit 1:500; Alomone), Kv7.4 (anti-Rabbit 1:200; Neuromab) or Kv7.5 (anti-Rabbit 1:500; Abcam) for 1 h 15 min (Supplemental Table II). The cells were washed three times with PBS and incubated for 2 h in the dark with the secondary antibody (Donkey anti-Mouse/Rabbit Alexa Fluor 555; Invitrogen, UK) at 1:1000 dilution. Cells were then washed three times with PBS and mounted onto glass microscope slides and sealed. DAPI (4′,6-diamidino-2-phenylindole) was used in the mounting medium (Vectashield) to stain the nuclei. Cells were imaged using inverted confocal microscope LSM880 (Zeiss) with a 40x objective at wavelength 405 nm (DAPI) and 555 nm (antibody). Zeiss Zen software and Fiji ImageJ software were used to produce images.

### 2.5 HEK293 cell culture and transfection

Human embryonic Kidney line 293 cells were cultured to 80 % confluency before passaging and used for experimentation between P10 and P40. Cells were grown in DMEM (Gibco Life Sciences, UK) containing penicillin (100 U/mL), streptomycin (100 μg/mL) and 10 % FBS. For patch clamp electrophysiology HEK293 cells were cultured in 24-well plates for 24 h prior to transfection, transfected with 400 ng of eYFP tagged *KCNQ4* (AF105202) or untagged *KCNQ3, KCNQ5* (1:1; these plasmids were kindly provided by Mark Shapiro, University of Texas Health Science Center at San Antonio, Texas, USA) together with eYFP. FuGene® HD (Promega, UK) was used for transfection, according to the instructions of manufacturer. Cells were treated with reaction mix in DMEM for 16−18 h before being washed from the wells, pelleted and re-plated onto 10mm glass coverslips at varying dilutions. eYFP was used for cell detection.

### 2.6 Voltage clamp electrophysiology

*KCNQ*/eYFP transfected HEK293 cells were used to isolate the Kv7 potassium currents and study them independently of other ion channels. Kv7 current was recorded using a HEKA EPC10 amplifier and Patchmaster V2 software (HEKA instruments) using an inverted step protocol where cells were held at −20 mV, and 600 ms deactivating pulses to −60 mV were applied with 2.5 s interval. To investigate current-voltage relationships a standard IV protocol from −80 mV to +40 mV in 10 mV increments with a deactivating pulse to −70 mV was used. In this protocol, the tail current elicited by the step to −70 mV is measured to remove the impact of driving force on the recording. Pipettes were pulled using a horizontal puller (Sutter P-97) and fire polished to typically 2–4 MΩ resistance. Upon entering whole-cell configuration, cell capacitance was nulled. Experimental compounds were dissolved using DMSO as a vehicle in extracellular solution and control recordings were taken with DMSO present. Intracellular solution contained (in mM): 160 KCl, 5 MgCl_2_, 5 HEPES, 0.1 BAPTA, 3 K-ATP, 0.1 GTP; pH adjusted to 7.4 using NaOH. Extracellular solution contained (in mM): 160 NaCl, 2.5 KCl, 1 MgCl_2_, 10 HEPES, 2 CaCl_2_ and 10 Glucose; pH adjusted to 7.4 with NaOH; osmolarity adjusted to 320 mOsm.

### 2.7 Chemicals and reagents

NNC 55-0396 and edelfosine were from Tocris Bioscience (UK); XE991, retigabine, nifedipine, AVP, 2-Aminoethoxydiphenyl borate (2-APB), tetracaine and all other chemicals were obtained from Sigma-Aldrich (UK) (Supplemental Table III).

### 2.8 Statistical analysis

Fura2 and RT-PCR results are expressed as means ± S.E.M. Statistical comparisons were made using paired or unpaired student’s *t*-test or ANOVA followed by a Sidak post hoc, as appropriate. Voltage clamp recordings were quantified using Fitmaster V2 (HEKA instruments) by measuring the tail current from each voltage step immediately after the return step to −70 mV. Fitting the tail currents with a Boltzmann equation showed a sigmoidal current voltage relationship as expected for voltage dependent potassium channels. Repeated measures ANOVA or 2-tailed *t*-test were used to assess differences between treatment groups. *P* values less than 0.05 were considered to indicate a significant difference between the groups. Statistical analysis was performed using GraphPad Prism 7.

## 3 Results

### 3.1 Contribution of L-and T-type Ca^2+^ channels to depolarisation-induced Ca^2+^ transients in A7r5 cells

**Fig. 1:**
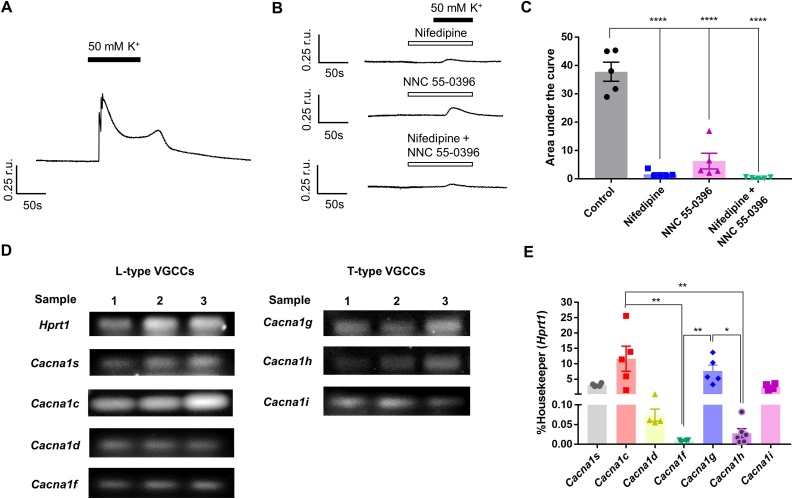
Contribution of L-and T-type VGCCs to depolarisation-induced Ca^2+^ transients in A7r5 cells. (A) Representative example trace showing rises in [Ca^2+^]_i_ (indicated as F340/F380 ratio units; r.u.) evoked by depolarising cells with 50 mM K^+^-containing buffer (the period indicated by the solid bar). (B) Example traces of high-K^+^-induced Ca^2+^ transients recorded in the presence of L-type (nifedipine; 2 μM) or T-type (NNC 55-0396; 3 μM) Ca^2+^ channel blockers (as indicated). (C) Bar graph showing the mean area under the curve of the response to 50 mM K^+^ buffer (control group represented in panel A) and cell groups in the presence of nifedipine or NNC 55-0396 (represented in panel B). (D) Agarose gels stained with SYBR safe to visualise the RT-PCR products corresponding to L-type (*Cacna1s*, *Cacna1c*, *Cacna1d*, *Cacna1f*) and T-type (*Cacna1g*, *Cacna1h*, *Cacna1i*) VGCCs genes. (E) Quantification of RT-PCR results exemplified in panel D; expression is normalised to that of a housekeeping gene, *Hprt1* (hypoxanthine phosphoribosyltransferase 1). In panels C and E data are presented as mean ± S.E.M.; *P < 0.05, **P < 0.01, ****P < 0.0001 (panel C, n = 5; panel E, n≥4).

We reasoned that since Kv7 channels control the resting *E*_m_ of cells and because these channels are voltage-gated, they may exert a degree of control over the Ca^2+^ influx through the VGCCs. Thus, as a first step, we investigated the contribution of the most abundantly expressed VGCC isoforms to the depolarisation-induced Ca^2+^ influx in A7r5 rat smooth muscle cells. Depolarisation was produced with perfusion with a ‘High-K^+^’ solution, which was similar to the standard bath solution but contained 50 mM K^+^ (produced by the equimolar substitution of NaCl with KCl). Fig.1A shows an example of a response to 50 mM K^+^ buffer in A7r5 cells: a rapid transient increase in [Ca^2+^]_i_ followed by a slow decline, presumably due to VGCCs inactivation and extrusion of Ca^2+^ from cytosol by Ca^2+^ ATPases and/or Na^+^/Ca^2+^ exchanger. A limited number of rapid oscillations was often observed during the transient. Smooth muscle cells mainly express L- and T-type VGCCs, while other isoforms are not significantly expressed [33,34]. Accordingly, nifedipine (2 μM), L-type Ca^2+^ channel blocker [35] and, NNC 55-0396 (3 μM), a structural analogue of mibefradil which selectively inhibits T-type Ca^2+^ channels [36], suppressed the amplitude and reduced the area under the curve (AUC) of high-K^+^-induced [Ca^2+^]_i_ transients by 95.9 ± 2.3 % (n = 5) and by 85.0 ± 13.3 % (n = 5), respectively (Fig. 1B,C). Nifedipine-induced inhibition was somewhat stronger than that produced by NNC 55-0396, but the effect did not reach statistical significance. Co-application of both inhibitors completely abolished the high-K^+^-induced Ca^2+^ transients (Fig. 1B,C). This suggested that both L- and T-type Ca^2+^ channels contribute to the depolarisation-induced Ca^2+^ influx in A7r5 cells, with L-type channels being the functionally predominant isoform for high K^+^-induced [Ca^2+^]_i_ transients. The additivity of the nifedipine and NNC 55-0396 effects perhaps arose from less than perfect selectivity as some inhibition of T-type Ca^2+^ channels by micromolar nifedipine has been reported [37] and, similarly, residual effect of NNC 55-0396 on native L-type Ca^2+^ channels cannot be excluded. Endpoint and RT-PCR were performed to evaluate the relative mRNA abundance of genes coding for L-type (*Cacna1s*, *Cacna1c*, *Cacna1d*, *Cacna1f;* coding for Cav1.1, Cav1.2, Cav1.3 and Cav1.4, respectively) and T-type (*Cacna1g*, *Cacna1h*, *Cacna1i*; coding for Cav3.1, Cav3.2 and Cav3.3, respectively) VGCC subunits. We found that *Cacna1c* (Cav1.2) L-type and *Cacna1g* (Cav3.1) T-type Ca^2+^ channel genes were the predominant subtypes in A7r5 cells (Fig. 1D,E).

### 3.2 Kv7 channel inhibition induces Ca^2+^ oscillations linked to L-and T-type VGCCs activity

**Fig. 2:**
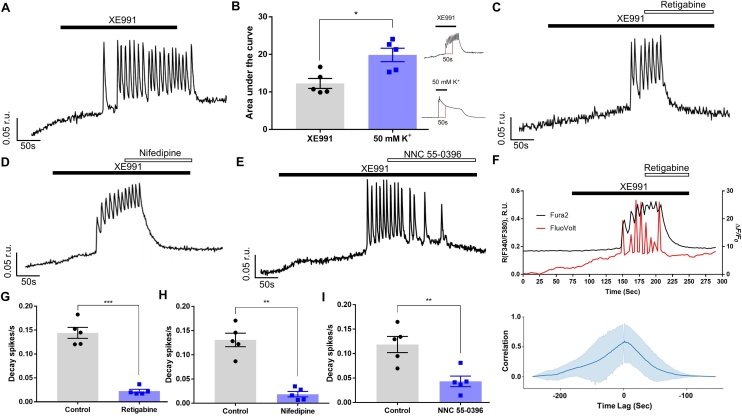
Kv7 channel inhibition induces Ca^2+^ oscillations linked to L- and T-type VGCCs activity. (A) Representative example trace showing Ca^2+^ oscillations (indicated as F340/F380 ratio units; r.u.) evoked by Kv7 channel inhibitor XE991 (10 μM). (B) Comparison of amplitude of Ca^2+^ signals induced by XE991 and 50 mM K^+^, estimated as area under the curve during first 50 s of stimulus application (corresponding to the duration of high-K^+^ stimulation; exemplified in the inset on the right). (C-E) Example traces of XE991-induced Ca^2+^ transients recorded in the presence of Kv7 channel opener, retigabine (10 μM; C), L-type (nifedipine; 2 μM; D) or T-type (NNC 55-0396; 3 μM; E) Ca^2+^ channel blockers (as indicated). (F) Upper panel: superimposed are Fura2 ratiometric Ca^2+^ recording (black) and FuoVolt membrane potential recording (measured as ΔF/F_0_; red) during application of XE991 (10 μM) and retigabine (10 μM) during periods indicated by horizontal bars. Lower panel: Cross correlation of the normalised ratiometric Ca^2+^ signal with the normalised FluoVolt signal indicated the time lag between the signals at which the peak correlation occurred. (G-I) Bar graphs summarising the effects of retigabine (G), nifedipine (H) or NNC 55-0396 (I) on the XE991-induced Ca^2+^ spike frequency (spikes/s). Control is the spike frequency in the presence of XE991 measured from the onset of the first spike. For quantification of drug effect spike frequency was calculated from the onset of the drug application and until the end of the application of XE991. In panels G-I data are presented as mean ± S.E.M.; **P < 0.01, ***P < 0.001 (n = 5).

We next tested if Kv7 inhibition would depolarise VSMCs and trigger Ca^2+^ influx. Exposure of A7r5 cells to a specific Kv7 channel inhibitor, XE991 (10 μM) induced a sustained [Ca^2+^]_i_ elevation and evoked Ca^2+^ oscillations persisting for the duration of XE991 application; both effects recovered upon XE991 washout (albeit with a delay of ∼50 s; Fig. 2A). Depolarisation with high-K^+^ caused larger [Ca^2+^]_i_ elevation than these produced by XE991 (Fig. 2B). We then applied Kv7 channel activator, retigabine, to test if it can potentiate Kv7 channel currents and hyperpolarise the *E*_m_ away from the activation threshold of VGCCs [38,39]. Retigabine (10 μM) applied along with the XE991 promptly abolished the oscillations and lowered [Ca^2+^]_i_ back to the baseline (Fig. 2C,G). The high efficacy of retigabine to reverse the XE991-induced excitation suggests that at 10 μM XE991 does not completely block native Kv7 channels in A7r5 cells (see below). To test if the [Ca^2+^]_i_ increase and oscillations induced by XE991 required Ca^2+^ influx from the extracellular space via VGCCs, we used nifedipine and NNC 55-0396. In the first experiment, XE991 (10 μM) was applied first to induce oscillations, then nifedipine (2 μM) was applied in the continued presence of XE991. Nifedipine virtually abolished the oscillations and reverted [Ca^2+^]_i_, elevation induced by XE991 (Fig. 2D,H). NNC 55-0396 produced qualitatively similar effect but it took longer to block oscillations (Fig. 2E,I). Among three compounds, retigabine and nifedipine were the strongest to abolished Ca^2+^ oscillations (Fig. 2G,H). These data demonstrated that the XE991-induced [Ca^2+^]_i_ increase required Ca^2+^ entry via both L- and T-type Ca^2+^ channels.

We next asked what is the nature of [Ca^2+^]_i_, oscillations induced by XE991 - might these be induced by the depolarisation-induced action potentials? We loaded A7r5 cells with two fluorescent dyes: Fura2 for measuring Ca^2+^ levels and a voltage-sensitive dye FluoVolt [40] for measuring changes in membrane potential (*E*_m_). We then performed a triple-wavelength (340, 380 and 488 nm) simultaneous recordings of [Ca^2+^]_i_ and *E*_m_ changes in response to XE991. Fig. 2F displays temporally-aligned ratiometric Ca^2+^ trace (black) and FluoVolt *E*_m_ trace (red); XE991 induced slow *E*_m_ depolarisation followed by a burst of sharp spikes which were temporally well-correlated with [Ca^2+^]_i_ (upper panel). Peak cross correlation between the FluoVolt *E*_m_ and the ratiometric Ca^2+^ signal was 0.6 ± 0.18, occurring at a time lag of 3.0 ± 0.5 s (n = 5) (lower panel). Both, [Ca^2+^]_i_ oscillations and *E*_m_ spikes were abolished by retigabine.

**Fig. 3:**
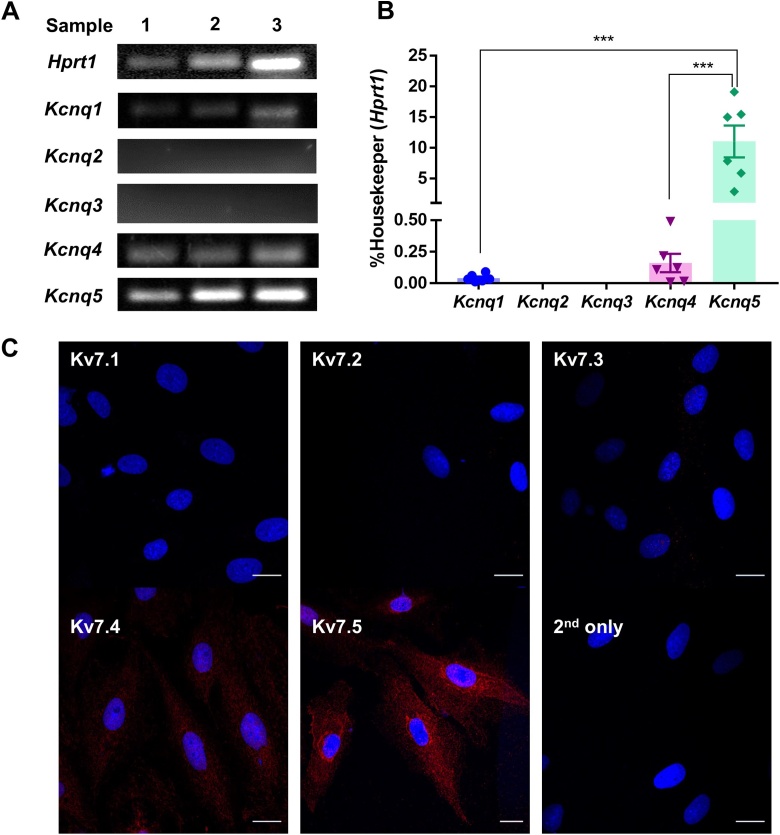
Expression of *Kcnq* genes and Kv7 proteins in A7r5 cells. (A) Agarose gels stained with SYBR safe to visualise the RT-PCR products of *Kcnq1*, *Kcnq2*, *Kcnq3*, *Kcnq4* and *Kcnq5*. (B) Quantification of RT-PCR results exemplified in panel A, expression is normalised to that of a housekeeping gene, *Hprt1* (hypoxanthine phosphoribosyltransferase 1). (C) Immunofluorescence labelling of Kv7.1 – Kv7.5 channel subunits in A7r5 cells, scale bars are 20 μm. In panel B data are presented as mean ± S.E.M.; ***P < 0.001 (n = 6).

**Fig. 4:**
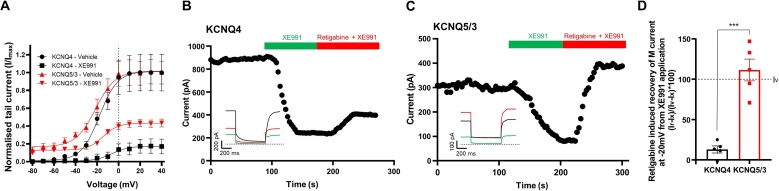
XE991 elicits a partial blockade of current through Kv7.5/Kv7.3 heteromeric channels that can be fully recovered by retigabine. (A) Current voltage relationship of Kv7.4 (n = 8) and Kv7.5/3 (n = 10) channels prior to and post XE991 (10 μM) treatment. (B,C) Representative voltage clamp recordings at −20 mV showing effects of XE991 (10 μM) and retigabine (10 μM; applied in the presence of XE991) on Kv7.4 (B) or Kv7.5/3) (C) channel currents. (D) Retigabine induced recovery (Ir) of Kv7 (M) current at −20 mV after XE991 application (Ix) expressed as a percentage of the control (vehicle) current (Iv). Recovery calculated as (Ir-Ix)/(Iv-Ix). Data are presented as mean ± S.E.M.; ***P < 0.001 (independent measures two-tailed *t*-test).

Next, we investigated the expression of *Kcnq* genes in A7r5 cells with RT-PCR using selective primers for *Kcnq1-5* isoforms. We detected transcripts for *Kcnq1*, *Kcnq4* and *Kcnq5,* with *Kcnq5* being the most and *Kcnq1* the least abundant (Fig. 3A,B). *Kcnq2* and *Kcnq3* transcripts were undetectable. Consistent with the RT-PCR results, we detected expression of Kv7.5 and Kv7.4, but not Kv7.1-Kv7.3 in A7r5 cells using immunohistochemistry (Fig. 3C); Kv7.5 was again found to be most abundantly expressed. These data were not statistically compared because the primary antibody affinities to their respective epitopes are not uniform and cannot be meaningfully normalised. The fact that *Kcnq5* was found to be the main *Kcnq* gene expressed in A7r5 cells is consistent with previous findings [15] and explains good reversibility of the excitatory action of 10 μM XE991 with retigabine. Indeed, while Kv7.1-Kv7.4 channels are highly sensitive to XE991 with IC_50_s in the range of 1–5 μM [[41], [42], [43]], Kv7.5 is much less sensitive with IC_50_ in the range of 60 μM [44]. Thus, while currents conducted by Kv7.2/Kv7.3 channels are completely abolished by 10 μM XE991 [41], Kv7.5 is inhibited by just ∼40 % at this concentration [45]. To test if this hypothesis is correct, we asked if retigabine would recover different cloned Kv7 channels from the XE991-induced inhibition. Since Kv7.5 expresses poorly on its own [46], we expressed it as a heteromeric channel together with Kv7.3 in HEK293 cells and compared the effects of 10 μM XE991 and 10 μM retigabine between the Kv7.3/Kv7.5 and Kv7.4 channels. At the concentration used, XE991 inhibited homomeric Kv7.4 by over 80 % while inhibition of Kv7.3/Kv7.5 heteromers was less than 60 % (Fig. 4A). Accordingly, when 10 μM retigabine was applied after (and still in the presence of) XE991, it produced only a marginal increase of the Kv7.4 steady-state current amplitude at −20 mV (Fig. 4B,D), while its effect on the Kv7.3/Kv7.5 current was much more prominent and such that the current amplitude of Kv7.3/Kv7.5 current was recovered above the basal level (before XE991 application; Fig. 4C,D). These experiments further support the notion that Kv7.5 is a major Kv7 subunit in rat VSMCs.

### 3.3 AVP-induced Ca^2+^ oscillations can be abolished by Kv7 activator or L-and T-type Ca^2+^ channel blockers

**Fig. 5:**
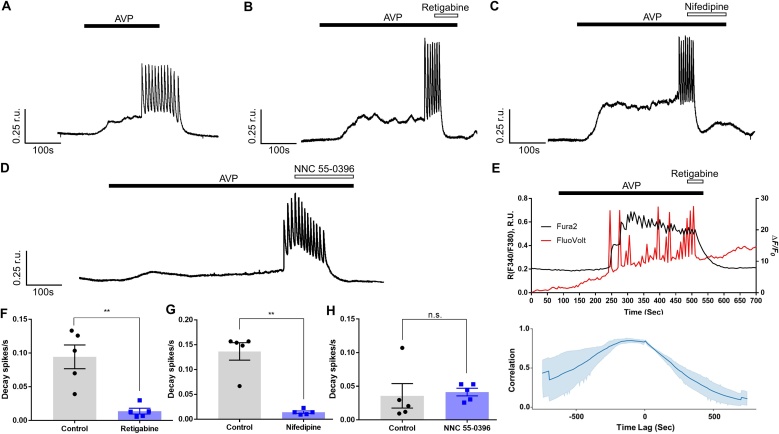
AVP-induced Ca^2+^ oscillations can be abolished by Kv7 activator, L- and T-type Ca^2+^ channel blockers. (A) Representative example trace showing Ca^2+^ oscillations (indicated as F340/F380 ratio units; r.u.) evoked by the physiological concentration of vasoactive hormone, vasopressin (AVP; 100 pM) in A7r5 cells. (B-D) Example traces of AVP-induced Ca^2+^ transients recorded in the presence of Kv7 channel opener, retigabine (10 μM; B), L-type (nifedipine; 2 μM; C) or T-type (NNC 55-0396; 3 μM; D) Ca^2+^ channel blockers (as indicated). (E) Upper panel: superimposed are Fura2 ratiometric Ca^2+^ recording (black) and FuoVolt membrane potential recording (measured as ΔF/F_0_; red) during application of AVP (100 pM) and retigabine (10 μM) during periods indicated by horizontal bars. Lower panel: Cross correlation of the normalised ratiometric Ca^2+^ signal with the normalised FluoVolt signal indicated the time lag between the signals at which the peak correlation occurred. (F-H) Bar graphs summarising the effects of retigabine (F), nifedipine (G) or NNC 55-0396 (H) on the AVP-induced Ca^2+^ spike frequency (spikes/s). Control is the spike frequency in the presence of AVP measured from the onset of the first spike. For quantification of drug effect spike frequency was calculated from the onset of the drug application and until the end of the application of AVP. In panels F-H data are presented as mean ± S.E.M.; n.s., not significant; **P < 0.01 (n = 5).

To investigate if the control over the *E*_m_ and [Ca^2+^]_i_ exerted by the Kv7 channels in VSMCs is relevant for the hormonal regulation of vascular tone, we used AVP (100 pM), which has been reported to inhibit KCNQ currents [15]. AVP is a hormone with a prominent vasoconstrictor effect, which acts in the vasculature primarily via V1AR receptor, a G_q/11_ type of GPCR which acts via the PLC pathway [23]. Exposure to AVP induced a slow [Ca^2+^]_i_ elevation followed by a period of repetitive Ca^2+^ oscillations lasting throughout the AVP application; washout of the AVP was followed by a gradual return to the baseline (Fig. 5A). Retigabine promptly abolished AVP-induced Ca^2+^ oscillations and reverted the elevated [Ca^2+^]_i_ to the baseline (Fig. 5B,F). To test the contribution of L- and T-type Ca^2+^ channels to the AVP-induced Ca^2+^ oscillations, we used nifedipine and NNC 55-0396. In the presence of nifedipine, AVP-induced Ca^2+^ oscillations were rapidly stopped, and the [Ca^2+^]_i_ returned to the basal level (Fig. 5C,G). In the presence of NNC 55-0396, AVP-induced Ca^2+^ oscillations were gradually stopped in a time-dependent manner (Fig. 5D,H). We also performed a triple-wavelength simultaneous recordings of [Ca^2+^]_i_ and *E*_m_ changes in response to AVP (Fig. 5E). Similar to XE991, AVP induced slow *E*_m_ depolarisation followed by a burst of sharp spikes which were temporally well-correlated with [Ca^2+^]_i_ (upper panel); peak cross correlation between the FluoVolt *E*_m_ and the ratiometric Ca^2+^ signals was 0.84 ± 0.01, occurring at a time lag of 2.5 ± 1.4 s (n = 4) (lower panel). These [Ca^2+^]_i_ oscillations and *E*_m_ spikes were abolished by retigabine. Collectively, these data indicate that Kv7/KCNQ channels are present and functional in A7r5 cells and that treatment with a physiological concentration of AVP (100 pM) leads to depolarisation and Ca^2+^ influx through both L- and T-type Ca^2+^ channels; these effects were abolished by retigabine and were qualitatively very similar to these produced by the XE991.

### 3.4 AVP-induced Ca^2+^oscillations are reduced by inhibition of PLC and ER Ca^2+^ release channels

**Fig. 6:**
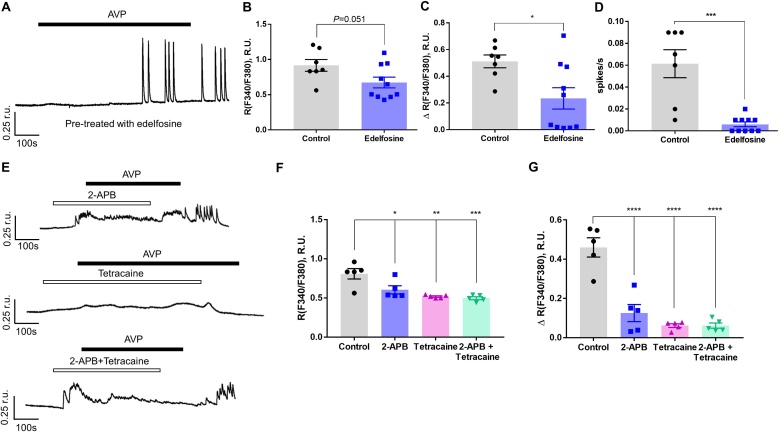
AVP-induced Ca^2+^ oscillations are reduced by inhibition of PLC and ER Ca^2+^ release channels. (A) Representative example trace showing Ca^2+^ oscillations (indicated as F340/F380 ratio units; r.u.) evoked by AVP (100 pM) in A7r5 cells pretreated with the phospholipase C (PLC) inhibitor, edelfosine (10 μM). (B-D) Bar graphs summarising the effects of edelfosine on the peak Ca^2+^ level (B), Ca^2+^ response amplitude (ΔR; C) and Ca^2+^ spike frequency (spikes/s) (D) induced by AVP. (E) Example traces of AVP-induced Ca^2+^ transients recorded in the presence of 2-APB (IP_3_Rs inhibitor; 100 μM) or tetracaine (RyRs inhibitor; 100 μM), as indicated. (F,G) Bar graphs summarising the effects of the effects of 2-APB or tetracaine on the peak Ca^2+^ level (F) and Ca^2+^ response amplitude (ΔR; G) induced by AVP. In panels B-D and F-G data are presented as mean ± S.E.M.; *P < 0.05, **P < 0.01, ***P < 0.001, ****P < 0.0001 (panel B-D, Control, n = 7, edelfosine, n = 10; panel F-G, n = 5).

Even though both XE991 and AVP produced very similar effects on [Ca^2+^]_i_ and *E*_m_, suggesting a common mechanism, a Kv7 channel inhibition, the action of these two agents on the Kv7 channels is different. While XE991 is a direct ion channel blocker, the action of AVP on the Kv7 channels depends on the PLC-mediated signalling cascade, which inhibits Kv7s by a combination of PIP_2_ hydrolysis, PKC activation and ER Ca^2+^ release [12]. Edelfosine is a PLC inhibitor that is used as a test for the involvement of PLC in a signalling pathway [47]. Ten min pretreatment of A7r5 cells with edelfosine (10 μM) attenuated the [Ca^2+^]_i_ response to AVP (100 pM) in A7r5 cells (Fig. 6A). There was marginally significant difference (*p* = 0.051) in peak Ca^2+^ levels between the control (untreated) and edelfosine-treated groups (Fig. 6B). There were some occasional Ca^2+^ spikes but the Ca^2+^ response and spike frequency were significantly reduced (Fig. 6C,D).

**Fig. 7:**
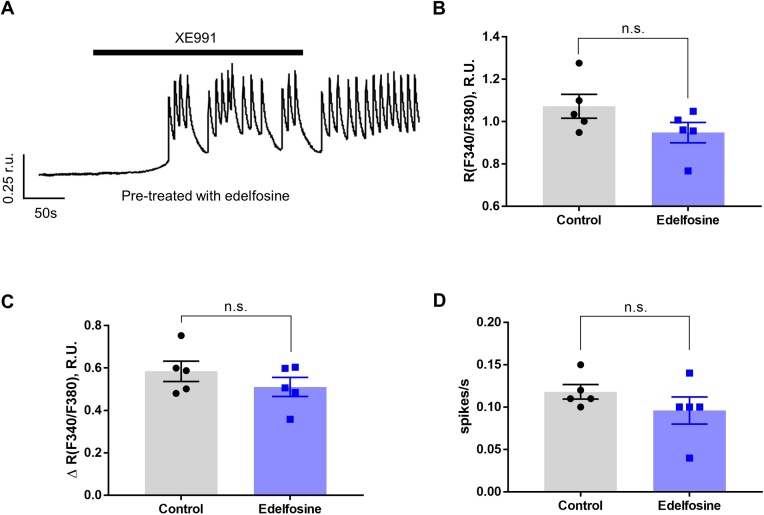
Ca^2+^ oscillations induced by Kv7 channel inhibition are insensitive to PLC inhibition. (A) Representative example trace showing Ca^2+^ oscillations (indicated as F340/F380 ratio units; r.u.) evoked by XE991 (10 μM) in A7r5 cells pretreated with PLC inhibitor, edelfosine (10 μM). (B-D) Bar graphs summarising the effects of edelfosine on the peak Ca^2+^ level (B), Ca^2+^ response amplitude (ΔR; C) and Ca^2+^ spike frequency (spikes/s) (D) induced by XE991. In panels B-D data are presented as mean ± S.E.M.; n.s., not significant; (n = 5).

Since PLC activation produces IP_3_ and triggers ER store release, we next analysed the contribution of IP_3_Rs and RyRs to the AVP-induced Ca^2+^ signals in A7r5 cells using 2-APB or tetracaine, respectively. Pretreatment with 2-APB (2 min) significantly attenuated the amplitude of the sustained [Ca^2+^]_i_ elevation induced by AVP (100 pM); it did not completely abolished oscillations but reduced their amplitude. RyRs inhibitor, tetracaine, effectively inhibited the amplitude and frequency of Ca^2+^ oscillation caused by AVP, and when applied together, 2-APB and tetracaine significantly attenuated [Ca^2+^]_i_ response to AVP (Fig. 6E-G). We next tested if PLC is required for the Ca^2+^ oscillations evoked by Kv7 channel inhibitor. A7r5 cells were pre-treated (10 min) with edelfosine and the effect of XE991 (10 μM) was explored with Ca^2+^ imaging. Edelfosine (10 μM) did not inhibit Ca^2+^ oscillations: neither the amplitude of sustained [Ca^2+^]_i_ elevation nor spike frequency were different from control (Fig. 7). These data indicate that PLC activation and ER Ca^2+^ release are the necessary factors linking AVP to Kv7 channel inhibition in A7r5 cells. At the same time, Ca^2+^ response triggered by direct inhibition of Kv7 channels with XE991 did not require PLC activation. It must be noted that edelfosine, 2-APB and tetracaine are not entirely selective for their targets. Thus, edelfosine, in addition to inhibiting PLC, also activates platelet-activating factor (PAF) receptors [48]. While 2-APB inhibits the IP_3_Rs without an effect on the RyRs [49], it also inhibits TRPC3, 6 and 7 channels in VSMCs [50]. Tetracaine, a RyR antagonist which is used to eliminate RyR-dependent Ca^2+^ sparks [51,52] is also reported to influence Kv channels [53]. Thus, caution is needed in interpreting the results presented in Fig. 6, Fig. 7.

### 3.5 AVP-induced Ca^2+^ transients in human IMA SMCs can be inhibited by Kv7 activator and Ca^2+^ channel inhibitors

**Fig. 8:**
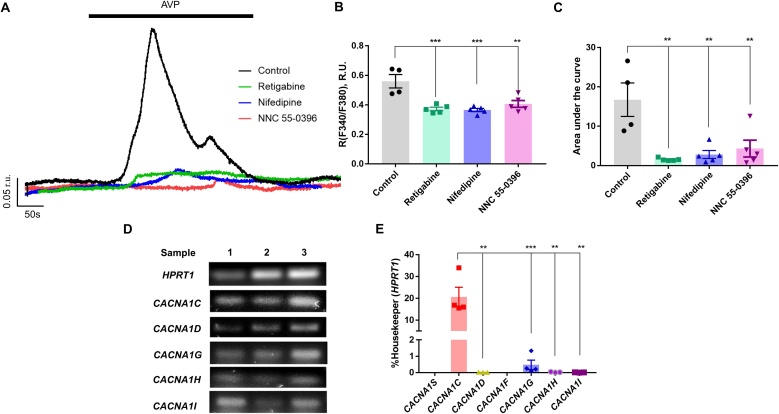
AVP-induced Ca^2+^ transients in human IMA SMCs is reduced by Kv7 activator, L- and T-type Ca^2+^ channel blockers. (A) Representative example traces showing rises in [Ca^2+^]_i_ (indicated as F340/F380 ratio units; r.u.) evoked by AVP (100 pM; the application period is indicated by the solid bar) in control conditions (black) or in the presence of Kv7 channel opener, retigabine (10 μM; green), L-type (nifedipine; 2 μM; blue) or T-type (NNC 55-0396; 3 μM; red) Ca^2+^ channel blockers (as indicated). (B,C) Bar graphs showing the peak Ca^2+^ level (B) and mean area under the curve of the response (C) to AVP. (D) Agarose gels stained with SYBR safe to visualise the RT-PCR products corresponding to L-type (*CACNA1C*, *CACNA1D*) and T-type (*CACNA1 G*, *CACNA1H*, *CACNA1I*) VGCCs genes. (E) Quantification of RT-PCR results exemplified in panel D, expression is normalised to that of a housekeeping gene, *HPRT1* (hypoxanthine phosphoribosyltransferase 1). In panels B, C and E data are presented as mean ± S.E.M.; **P < 0.01, ***P < 0.001 (panels B,C, n≥4; panel E, n≥3).

**Fig. 9:**
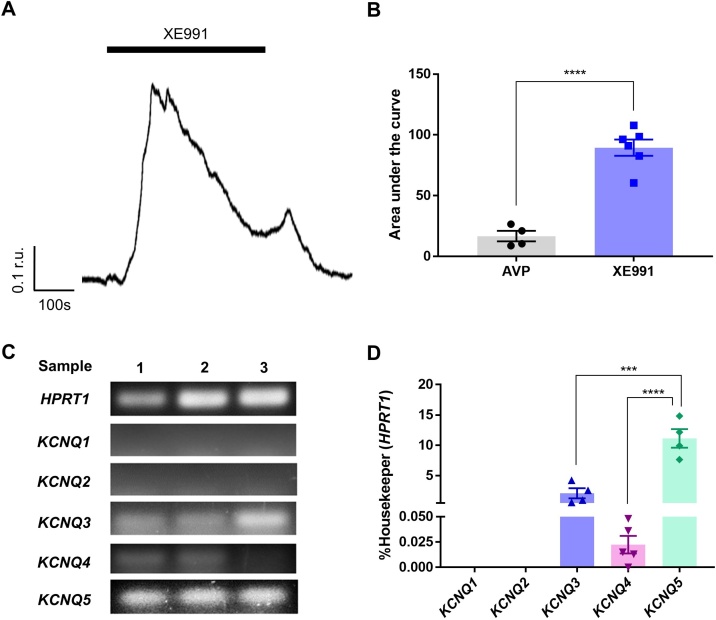
Functional expression of Kv7 channels in primary human IMA cells. (A) Representative example trace showing rises in [Ca^2+^]_i_ (indicated as F340/F380 ratio units; r.u.) evoked by XE991 (10 μM; the application period is indicated by the solid bar). (B) Comparison of Ca^2+^ signals (area under the curve) induced by AVP and XE991 in IMA cells. (C) Agarose gels stained with SYBR safe to visualise the RT-PCR products of *KCNQ1*, *KCNQ2*, *KCNQ3*, *KCNQ4* and *KCNQ5*. (D) Quantification of RT-PCR results exemplified in panel C, expression is normalised to that of a housekeeping gene, *HPRT1* (hypoxanthine phosphoribosyltransferase 1). In panels B and D data are presented as mean ± S.E.M.; ***P < 0.001, ****P < 0.0001 (n≥4).

Next, we tested how Kv7 channel activity affects AVP-induced Ca^2+^ signalling in primary human IMA SMCs (see Methods). AVP (100 pM) caused a sharp increase of the [Ca^2+^]_i_, however, there were no oscillations (Fig. 8A). Retigabine applied 2 min before and during administration of AVP almost completely abolished the AVP-induced increase of [Ca^2+^]_i_ in human IMA SMCs. Either nifedipine or NNC 55-0396 applied in a similar way also significantly reduced the [Ca^2+^]_i_ transients (Fig. 8A-C), with retigabine being most efficacious of the three compounds tested (Fig. 8B,C). Endpoint and RT-PCR confirmed that *CACNA1* (Cav1.2) L-type and *CACNA1 G* (Cav3.1) T-type Ca^2+^ channel genes were the predominant subtypes in IMA SMCs (Fig. 8D,E). Although Kv7 channels have been reported in VSMCs of different origins [24,54] to our knowledge there is no such information for IMA SMCs. We, thus, investigated the function and expression of Kv7 channels in IMA SMCs. Application of XE991 (10 μM) induced a significant transient increase of [Ca^2+^]_i_ in IMA SMCs (Fig. 9A). Depolarisation with the inhibition of Kv7s caused larger [Ca^2+^]_i_ elevation than that produced by AVP (Fig. 9B). We detected transcripts for *KCNQ3*, *KCNQ4* and *KCNQ5,* but not *KCNQ1* or *KCNQ2,* with *KCNQ5* being the most abundant in IMA SMCs (Fig. 9C,D). Collectively, these data indicated that Kv7 channels were functional in human IMA SMCs. Pharmacological activation of KCNQ channels can effectively halt AVP evoked [Ca^2+^]_i_ signals in human arterial SMCs.

## 4 Discussion

Our study establishes the role for Kv7 channels in control over the intracellular Ca^2+^ signalling in rat and human VSMCs. First, we show that in A7r5 cells inhibition of endogenous Kv7 channels is sufficient to depolarise the membrane potential to trigger *E*_m_ spikes, mirrored by [Ca^2+^]_i_ oscillations. Kv7.5 and Kv7.4 were found to be the main Kv7 subunits expressed in A7r5 VSMCs, with Kv7.5 expressed at highest levels. A low level of *Kcnq1* transcript was found in A7r5 cells by RT-PCR but Kv7.1 expression was undetectable by immunohistochemistry. These findings are consistent with previous studies which demonstrated that various levels of *Kcnq1*, *Kcnq4* and *Kcnq5* were expressed in SMCs of different origins [11]; *Kcnq1* was the most abundant in a mouse portal vein [55]. Some studies showed higher levels of *Kcnq4*, followed by *kcnq1* and *kcnq5* in vascular myocytes [24,[56], [57], [58]]. However, another two studies found that in A7r5 cells only *Kcnq5* was detectable [15,16]. Interestingly, in our study [Ca^2+^]_i_ elevations induced by bulk depolarisation with high-K^+^ were larger than these produced by XE991 and contained much less oscillations; we hypothesise that this lack of continuous spiking is due to Ca^2+^ overload during sustained strong depolarisation produced by the high-K^+^ solution, an effect which is not recapitulated by Kv7 channel inhibition. Indeed, according to the Nernst formalism, elevation of extracellular K^+^ to 50 mM would shift *E*_K_ to near −25 mV and reset the *E*_m_ accordingly. Inhibition of Kv7 channels with XE991 is expected to produce milder depolarisation. Thus, depolarisation by ∼8 mV in response to 3 μM XE991 was reported in sensory neurons [59]. Since in A7r5 cells the main Kv7 subunit is Kv7.5, which is by far the least sensitive to XE991 Kv7 subunit [41,44], even at 10 μM XE991 (as used in the present study), and even with contribution of Kv7.4, only a partial inhibition of compound Kv7 current in A7r5 is expected. This would result in much less pronounced depolarisation, as compared to that produced by 50 mM extracellular K^+^.

Second, we show that the depolarisation-induced [Ca^2+^]_i_ raises and the XE991-induced Ca^2+^ oscillations were dependent on Ca^2+^ influx via the L- and T-type VGCCs. There are two important classes of VGCCs in VSMCs: the high voltage-activated (HVA) L-type Ca^2+^ channels and low voltage-activated (LVA) T-type Ca^2+^ channels [60,61]. Smooth muscle contractility is mainly dependent on an increase of [Ca^2+^]_i_ through the L-type Ca^2+^ channels [62]. We found that *Cacna1c*, coding for Cav1.2 L-type subunit and *Cacna1g*, coding for Cav3.1 T-type subunit were the predominant L- and T-type channel α-subunit transcripts expressed in A7r5 cells. A decrease of Kv channel activity either by the direct inhibition of the channel activity or by reduced expression can trigger an influx of Ca^2+^ via L-type Ca^2+^ channels [63,64]. Therefore, blockade of Kv7 channels with XE991 in VSMCs may induce *E*_m_ depolarisation and trigger action potentials and Ca^2+^ influx via the L-type Ca^2+^ channels ([6]; present study). We also report that the Ca^2+^ rises induced by XE991 were reversed by T-type Ca^2+^ blocker, NNC 55-0396, which abolished the Ca^2+^ oscillations gradually but completely. This could be explained by the contribution of low-threshold T-type Ca^2+^ channels to the spike initiation [65,66], such that T-type channel inhibition could reduce or block spike generation. However, a cross-inhibition of L-type Ca^2+^ channels by NNC 55-0396 (and also a degree of inhibition of T-type channels by nifedipine) cannot be presently excluded. Importantly, XE991-induced oscillations of both, *E*_m_ and [Ca^2+^]_i_ were completely blocked by the Kv7 opener, retigabine, indicating a strong control over the excitability and Ca^2+^ signalling in VSMCs exerted by the Kv7 channels. The inhibitory effect of retigabine was even stronger than that of the L-type VGCC blocker nifedipine, which is widely prescribed to treat cardiovascular disease [67]. Thus, Kv7 openers may have a potential clinical use, i.e. to promote vasodilatation.

Third, we show that the vasoconstrictor hormone AVP exerts effects on *E*_m_ and [Ca^2+^]_i_ that are very similar to these of XE991. Indeed, the main mechanism of action of such hormones to stimulate rhythmic constrictions of arteries has been recognized as depolarisation of the *E*_m_ to open VGCCs [23]. It has been shown that in A7r5 cells, physiological concentration of AVP (100 pM) leads to activation of PLC and PKC followed by inhibition of an outward voltage-sensitive K^+^ current (*I*_Kv_), which in turn, depolarised *E*_m_ to activate L-type Ca^2+^ channels and produce repetitive Ca^2+^ spiking, an effect which appeared to be mediated by Kv7.5 [15,68]. It has to be aknowledged that the AVP-induced Ca^2+^ signalling in SMCs is complex and may differ mechanistically between the SMC types. Thus, Ca^2+^-induced Ca^2+^ release via RyRs [69] and DAG-sensitive TRPC6 channels were also reported to contribute to AVP-induced membrane depolarisation and Ca^2+^ oscillations at physiological concentrations of AVP (10–100 pM) in A7r5 cells [70,71]. Moreover, activation of the RhoA/ROCK pathway by AVP was reported to stimulate nonselective cation influx (TRPC channels) via DAG-PKC signalling in VSMCs [72]. RhoA/ROCK pathway can also interfere with Ca^2+^ signalling through VGCCs contributing to the control of SMCs contraction and relaxation. Thus, inhibition of VGCC-mediated Ca^2+^ influx in VSMCs by Rho kinase inhibitors has been reported [73], however, the mechanisms linking RhoA/ROCK requres further investigation.

While the complexity of the receptor-mediated Ca^2+^ signaling in VSMCs should not be underestimated, our data suggests that AVP-mediated Kv7 inhibition, subsequent depolarisation and activation of L- and T-type Ca^2+^ channels strongly contribute to AVP signaling in A7r5 and human IMA cells. Specifically, we found that AVP mimics XE991 to generate Ca^2+^ spiking in A7r5 cells: both compounds depolarised *E*_m_ (as measured with FluoVolt) and induced [Ca^2+^]_i_ oscillations. The effect of AVP took longer to develop but resulted in a similar spike frequency to that of XE991 (Fig. 5). Effects of both compounds were blocked by VGCC inhibitors and retigabine.

Yet, there is an important difference between the way AVP and XE991 act upon Kv7 channels with former being a GPCR ligand acting via intricate intracellular signalling cascade, while the latter being a direct ion channel inhibitor. G protein-coupled vasoconstrictor agonist can induce early (transient) and late (sustained) phases of constriction via IP_3_-mediated Ca^2+^ release and Ca^2+^ entry from L-type Ca^2+^ channels, respectively [25]. Higher AVP concentrations (>1 nM) can stimulate the activation of PLC, which results in the production of IP_3_ and release of intracellular Ca^2+^ in A7r5 cells [25,74]. PLC-mediated inhibition of Kv7 channels is one of the signature effects of G_q/11_-coupled GPCR [75]. Within the multiple branches of this well-studied signalling cascade, PIP_2_ depletion [18,19], IP_3_-mediated ER Ca^2+^ release (in concert with calmodulin) [[76], [77], [78]] and activation of PKC (following the release of DAG, a product of PIP_2_ hydrolysis) [[79], [80], [81]] are the major events that can independently produce Kv7 channel inhibition. In reality though all three mechanisms usually act in concert, although contribution of each individual mechanism to the total Kv7 inhibition varies between cell and tissue types dramatically [12]. Our findings suggest that even at fairly low concentration of 100 pM, AVP mediates the recruitment of the PLC-IP_3_ signal transduction mechanism to induce Ca^2+^ spiking in A7r5 cells; the Kv7 channel inhibition is a likely contributor to this effect. Thus, a PLC inhibitor attenuated the amplitude of [Ca^2+^]_i_ elevation and mean spike frequency induced by AVP. Moreover, inhibition of IP_3_Rs and RyRs with 2-APB and tetracaine, respectively, also attenuated the AVP-induced Ca^2+^ oscillations (Fig. 6), suggesting that ER-released Ca^2+^ contributes specifically to Kv7 channel inhibition in A7r5 cells, as was suggested earlier for neurons [20,76]. As pointed earlier, that IP_3_R/RyR pharmacology is far from specific and further research will be recurred to specifically test contribution of IP_3_R/RyR to Ca^2+^ oscillations observed in this study. Importantly, AVP does not directly affect either L- and T-type Ca^2+^ channels currents in A7r5 cells [74], suggesting both these VGCCs are engaged indirectly, e.g. via the depolarisation produced by the Kv7 channel inhibition. While the fact that retigabine inhibits AVP responses does not explicitly prove that AVP-induced depolarisation and Ca^2+^ oscillations are mediated by Kv7 channel inhibition, the similarity of the XE991 and AVP effects, the dependence of the AVP action on PLC activity, and the wealth of data on strong inhibition of Kv7 channels by PLC-coupled GPCR do suggest that AVP-mediated Kv7 inhibition is a strong contributor the observed effects of AVP on *E*_m_ and [Ca^2+^]_i_ in A7r5 VSMCs.

Finally, we present evidence that Kv7 channel activity exerts a strong control over the intracellular Ca^2+^ signalling in primary human VSMCs as well. AVP has marked vasoconstrictor effect and gained attention as a possible tool against septic shock. However, it has been reported to induce graft vessel spasm during open-heart surgery [27]. Here we show that AVP causes a substantial increase of [Ca^2+^]_i_ which may be a characteristic feature in human IMA SMCs. Importantly, nifedipine, NNC 55-0396 and retigabine all significantly supressed the AVP-induced [Ca^2+^]_i_ transients, with retigabine being the most efficacious. Notably, there were no obvious oscillations in in human IMA SMCs in response to AVP or XE991. The reason for this difference between A7r5 and human primary SMCs cells remains to be elucidated, but it could be due to the more pronounced depolarisation and Ca^2+^ overload in the latter cell type. Despite this difference, these results indicated that Kv7 channels may have important clinical implications in cardiovascular disease and retigabine or its successors (e.g. drugs tailored to selectively activate Kv7.5) could have a potential in containment of the AVP-related perioperative vasospasm.

## CRediT authorship contribution statement

**Yuan-Ming Tsai:** Conceptualization, Methodology, Software, Validation, Formal analysis, Investigation, Visualization, Writing - original draft. **Frederick Jones:** Methodology, Software, Validation, Formal analysis. **Pierce Mullen:** Software, Formal analysis. **Karen E. Porter:** Conceptualization, Resources, Writing - review & editing. **Derek Steele:** Methodology, Writing - review & editing, Supervision. **Chris Peers:** Conceptualization, Methodology, Resources, Supervision. **Nikita Gamper:** Conceptualization, Methodology, Supervision, Resources, Funding acquisition, Writing - review & editing, Supervision.

## Declaration of Competing Interest

The authors have no conflicts of interest to report pertaining to this study.
