# Supplementary material for: Vascular Kv7 channels control intracellular Ca2+ dynamics in smooth muscle
Source: Cell Calcium. 2020 Dec;92:102283. doi: 10.1016/j.ceca.2020.102283 (PMC7695684; doi:10.1016/j.ceca.2020.102283)
Supplement: Supplementary file 1 [file mmc1.docx]

**Supplemental Tables**

**Supplemental Table I. Primers used for RT-PCR analysis of Kv7 and VGCCs expression**

| **Rat Primer** | **Assay ID** | **Human Primer** | **Assay ID** |
| --- | --- | --- | --- |
| Housekeeping gene | | | |
| *Hprt1* | Rn01527840_m1 | *HPRT1* | Hs02800695_m1 |
| L-type VGCCs | | | |
| *Cacna1s* | Rn01490941_m1 | *CACNA1S* | Hs00163885_m1 |
| *Cacna1c* | Rn00709287_m1 | *CACNA1C* | Hs00167681_m1 |
| *Cacna1d* | Rn01453395_m1 | *CACNA1D* | Hs00167753_m1 |
| *Cacna1f* | Rn00586734_m1 | *CACNA1F* | Hs00913770_m1 |
| T-type VGCCs | | | |
| *Cacna1g* | Rn01299126_m1 | *CACNA1G* | Hs00367969_m1 |
| *Cacna1h* | Rn01460348_m1 | *CACNA1H* | Hs01103527_m1 |
| *Cacna1i* | Rn01505208_m1 | *CACNA1I* | Hs01096207_m1 |
| Kv7 channels | | | |
| *Kcnq1* | Rn00583376_m1 | *KCNQ1* | Hs00923522_m1 |
| *Kcnq2* | Rn00591249_m1 | *KCNQ2* | Hs01548339_m1 |
| *Kcnq3* | Hs01120412_m1 | *KCNQ3* | Hs01120412_m1 |
| *Kcnq4* | Rn01518851_m1 | *KCNQ4* | Hs00542548_m1 |
| *Kcnq5* | Rn01512013_m1 | *KCNQ5* | Hs01068536_m1 |

**Supplemental Table II. Antibodies used for immunofluorescence labelling of Kv7.1 – Kv7.5 channel subunits**

| **Target antigen** | **Vendor or Source** | **Catalog #** | **Working**  **concentration** |
| --- | --- | --- | --- |
| Anti-KCNQ1 | Santa Cruz | SC-365186 | 1:500 |
| Anti-KCNQ2 | Santa Cruz | SC-271852 | 1:500 |
| Anti-KCNQ3 | Alomone | APC-051 | 1:500 |
| Anti-KCNQ4 | Neuromab | 75-082 | 1:200 |
| Anti-KCNQ5 | Abcam | ab19319 | 1:500 |

**Supplemental Table III. Drugs used in the experiment**

| **Name** | **Supplier** | **Catalog #** | **Solvent** | **Working Concentration** |
| --- | --- | --- | --- | --- |
| 2-APB | Sigma | D9754 | DMSO | 100 μM |
| AVP | Sigma | V9879 | Distilled water | 100 pM |
| Edelfosine | Tocris | 3022 | Distilled water | 10 μM |
| Nifedipine | Sigma | N7634 | DMSO | 2 μM |
| NNC 55-0396 | Tocris | 2268 | Distilled water | 3 μM |
| Retigabine | Sigma | SML0325 | DMSO | 10 μM |
| Tetracaine | Sigma | T7508 | Distilled water | 100 μM |
| XE991 | Sigma | X2254 | DMSO | 10 μM |
